# Surface Microstructure Regulation via Femtosecond Laser for Enhancing Laser Welding Strength of PVC/PA66: Mechanisms and Optimal Parameters

**DOI:** 10.3390/polym18111323

**Published:** 2026-05-27

**Authors:** Kehui Zhai, Fuyao Yang, Yu Lin, Minqiu Liu, Deqin Ouyang, Yewang Chen, Junqing Zhao, Qitao Lue, Shuangchen Ruan

**Affiliations:** 1 Key Laboratory of Advanced Optical Precision Manufacturing Technology of Guangdong Higher Education Institutes, Sino-German College of Intelligent Manufacturing, Shenzhen Technology University, Shenzhen 518118, China; zkh98210@163.com (K.Z.); yyangfuhao@163.com (F.Y.); 2450266002@stumail.sztu.edu.cn (Y.L.); ouyangdeqin@sztu.edu.cn (D.O.); chenyewang@sztu.edu.cn (Y.C.); zhaojunqing@sztu.edu.cn (J.Z.); lueqitao@sztu.edu.cn (Q.L.); scruan@sztu.edu.cn (S.R.); 2 College of Applied Technology, Shenzhen University, Shenzhen 518060, China; 3 Shenzhen Key Laboratory of Laser Engineering, Guangdong Provincial Key Laboratory of Micro/Nano Optomechatronics Engineering, College of Physics and Optoelectronic Engineering, Shenzhen University, Shenzhen 518060, China

**Keywords:** laser surface modification, laser transmission welding, surface microstructuring, mechanical interlocking, welding performance

## Abstract

The laser welding of incompatible polymers, such as polyvinyl chloride (PVC) and polyamide 66 (PA66), is often constrained by weak interfacial bonding, making it challenging to achieve joint strength that meets engineering requirements. This study proposes a welding reinforcement strategy based on femtosecond laser surface microstructuring regulation. First, high-precision and controllable microgroove structures were fabricated on the PVC surface, and the joint welding strength was significantly enhanced via the macroscopic mechanical interlocking effect. The influence of groove width, depth, spacing, and configuration on welding performance was systematically investigated. Subsequently, combined with fracture morphology characterization and finite element simulation, the interfacial reinforcement mechanism and stress regulation law of the microgroove structures were revealed. The results indicate that under the optimal process parameters (groove width = 70 μm, depth = 70 μm, spacing = 130 μm, welding power = 13 W), the joint with vertical groove structures achieves a maximum shear strength of 15.4 MPa, which is significantly superior to that of untreated joints. Additionally, vertical groove structures yield optimal unidirectional load-bearing strength, while grid groove structures effectively mitigate stress concentration under multidirectional loading, exhibiting better adaptability to complex stress conditions. This work provides a high-precision and versatile process for welding highly incompatible polymer systems, and also offers an important theoretical reference for process optimization and engineering applications of laser transmission welding of dissimilar polymers.

## 1. Introduction

Polymers and their composites, characterized by high specific strength, excellent fatigue resistance, and tunable biocompatibility, have become indispensable materials for lightweight aerospace components [1], implantable medical devices, and flexible electronics [2,3,4,5,6,7]. The composite assembly of dissimilar polymers is a key step in achieving the multi-functional integration of products. The current connection processes mainly include friction welding, ultrasonic welding, and laser welding. Among them, laser welding technology has become the mainstream technical solution for joining dissimilar polymers due to its advantages, such as a narrow heat-affected zone, high processing accuracy, and adaptability to complex structures. However, the inherent differences in the molecular structures of different polymers can lead to significant incompatibility between systems. As a result, during welding, the molten polymer molecules cannot fully diffuse and entangle, making direct welding prone to weak interfacial adhesion and low joint strength. Classic studies have confirmed that only 33 out of 342 common polymer pairs can be effectively blended, and the weldability of the majority of dissimilar polymer systems is extremely poor [8]. For instance, polyvinyl chloride (PVC) and polyamide 66 (PA66) are typical incompatible dissimilar polymer pairs. They have significant differences in molecular structure and poor interfacial compatibility. Moreover, the smooth welding surface lacks effective mechanical interlocking structures, making it difficult to achieve joint strength that meets engineering requirements through direct laser welding [9]. To improve the weldability of dissimilar polymers, current research mostly adopts surface modification methods before welding, including chemical grafting and plasma treatment [10,11,12]. Although these techniques, to a certain extent, can improve the interfacial connection strength of dissimilar polymers, their overall strength remains relatively low, as shown in Table 1. Moreover, most surface treatment methods are costly [13] and difficult to adapt to automated production line requirements.

Ultrafast laser surface etching technology can achieve high-precision and strongly controllable microstructure fabrication on material surfaces, while also regulating the chemical properties of the material surface [20,21,22]. It was first applied to the heterogeneous joining of polymers and metals in the lightweight field and has shown promising application potential in recent years [23,24]. However, it was not until 2023 that Liu et al. [25] first applied laser etching technology to the surface modification of polycarbonate (PC), and successfully achieved laser transmission welding of PC/PA66 with 30% glass fiber volume ratio (PA66GF30) based on the surface modification process. Laser etching not only induces the formation of a large number of polar functional groups on the PC surface, but also textures the micro-grooves required for the mechanical interlocking structure of the welding surface. The combination of enhanced polymer compatibility and the mechanical interlocking structure formed by the interfacial microstructure significantly improves the bonding strength. The maximum shear strength of the joint is more than 20 times higher than that of the pristine sample. In subsequent studies on femtosecond laser surface modification welding [9,19], the effects of femtosecond laser modification on polymer surfaces (such as wettability and chemical composition) were analyzed, providing additional chemical theoretical support for this method. Nevertheless, the current study only performed mechanical tests under the condition where the micro-slots are perpendicular to the tensile loading direction, without exploring the matching relationship between the groove orientation and the tensile loading direction, nor did it involve the performance research of complex structures such as grid grooves, thus failing to provide a design basis for multidirectional complex tensile loading conditions.

Research in the field of heterogeneous connections between polymers and metals has confirmed that the grid groove structure can effectively alleviate stress concentration under multidirectional loading and improve the adaptability of the joint to working conditions [26,27,28]. At the interface, the core strengthening mechanism of laser-etched microstructures for heterogeneous joints stems from the mechanical interlocking effect [29,30]. The molten materials to be joined can fill the microgrooves, and upon cooling and solidification, form a macroscopic anchoring structure. Under shear tensile loading, this anchoring structure can impede interfacial relative slip via mechanical interlocking, disperse the stress concentrated at the interface to the entire joint region, and concurrently inhibit the initiation and propagation of interfacial cracks, ultimately achieving an enhancement in joint strength. Grid microgrooves can form a stable anchoring structure along any tensile loading direction, effectively alleviating stress concentration under multidirectional complex loading and exhibiting superior working condition adaptability. However, the understanding of the aforementioned functioning rules and mechanical interlocking is based solely on the surface modification of rigid metals and polymer-based joining systems, and has not been proven in dissimilar polymer welding systems [27,31,32]. Meanwhile, current research on laser-etched welding of dissimilar polymers has not systematically established the relationship between microgroove geometric parameters and joint strength; process optimization mostly relies on empirical approaches, making it challenging to achieve precise regulation of microstructures and maximization of joint performance.

To address the aforementioned research gap, this study takes the dissimilar PVC/PA66 system as the research object and proposes a weldability-improving strategy based on femtosecond laser-induced surface microstructuring control. For the first time, the performance of three groove types—axial, vertical, and grid—was compared in a dissimilar polymer welding system. The stress distribution advantage of the grid structure under multidirectional loading was verified. Combined with ANSYS (2024.R2) finite element simulation, the intrinsic reasons for the superior strength of vertical grooves under uniaxial loading and the excellent multidirectional adaptability of grid grooves were explained, thereby improving the theoretical framework for femtosecond laser microstructuring to enhance polymer welding. Firstly, a femtosecond laser was employed to fabricate microgroove structures with varying geometric parameters and configurations on the PVC surface. Subsequently, a 1940 nm thulium-doped fiber laser was used to realize laser transmission welding of PA66 and PVC. The effects of groove width, depth, spacing, and configuration on joint shear strength were systematically investigated to determine the optimal process window, and a maximum joint strength of 15.4 MPa was ultimately achieved. Additionally, failed joint morphology characterization and finite element simulation were utilized to reveal the interfacial strengthening mechanism and stress regulation rule of the microgroove structures. The results indicate that the strengthening effect of grid microgrooves is not confined to unidirectional tensile loading, enabling adaptation to complex industrial application scenarios. This study provides a theoretical basis for process parameter optimization in laser transmission welding of dissimilar polymers and holds significant reference value for advancing the engineering application of laser welding technology.

## 2. Materials and Methods

### 2.1. Materials

PVC is widely applied in industrial and medical fields due to its favorable mechanical strength and chemical stability. In contrast, PA66, noted for its processability and excellent wear resistance, is regarded as a suitable material for manufacturing gears, bearings, automotive chassis parts, and medical devices. In this study, pure PVC and PA66 were selected as the base materials, both prepared with dimensions of 50 × 25 × 2 mm^3^.

### 2.2. Femtosecond Laser Surface Modification

**Figure 1:**
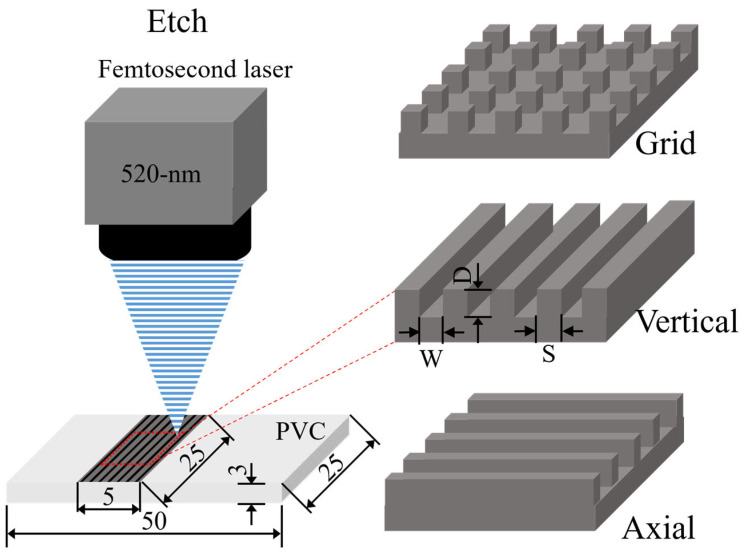
Schematic diagram of femtosecond laser etching.

Because PA66 exhibits a higher absorption rate for mid-infrared lasers than PVC, it melts earlier in the welding process. If microgrooves are fabricated on the PA66 surface, the melting behavior during welding will lead to the collapse of the groove structure, thereby failing to form an effective mechanical interlocking structure. In contrast, PVC exhibits a lower degree of melting during welding, allowing the etched microgroove morphology to be fully preserved. Therefore, PVC is used for laser surface etching to preserve the etched grooves. The laser surface treatment procedure is illustrated in Figure 1. The etching light source is a femtosecond laser with a wavelength of 520 nm, a maximum average output power of 8 W, a repetition rate of 250 kHz, a pulse width of 300 fs, a single-pulse energy of 32 μJ, a hatch spacing of 30 μm, and a pulse overlap rate of 50%. The laser’s output is controlled by a high-precision galvanometer system, and the spot diameter after focusing by the field lens is approximately 20 μm. The scanning speed is set at 2500 mm/s, and the process is repeated five times to ensure processing accuracy.

To investigate the influence of groove type (perpendicular axis, axial, and grid grooves), groove width (W), groove depth (D), and groove spacing (S) on subsequent welding strength, femtosecond laser etching was performed on the surface of PVC to produce grooves with varying parameters (Figure 1). The specific parameter levels are presented in Table 2. To maintain the surface chemical composition and microstructure, the etched PVC samples were cleaned by purging with compressed air at 0.25 MPa.

### 2.3. Laser Welding Experiment

**Figure 2:**
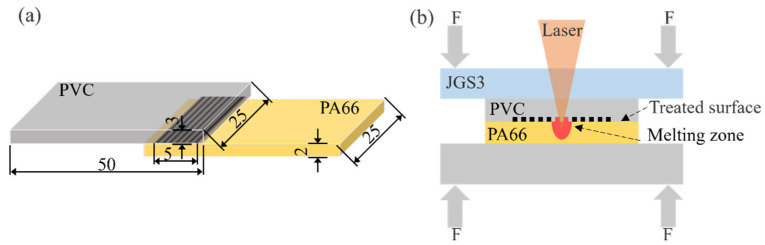
Experimental setup for laser welding: (**a**) Samples placed in lap joint configuration; (**b**) Laser welding experiment schematic diagram.

Most surface-pretreated samples were used in the welding experiments. The experiments were performed with a self-fabricated welding system equipped with a homemade 1940 nm continuous thulium-doped fiber laser as the light source. To investigate the effect of laser power (P) on welding quality, power levels ranging from 7.0 W, which was just sufficient to weld the samples under ablation conditions, to 17.0 W, which was just below the threshold for burning under etching conditions, were selected. The interval between experimental values was 2 W, resulting in six power levels. For each selected surface treatment condition or welding power level, at least three replicate experiments were carried out to evaluate the mechanical properties of the joints and to prepare weld cross-sections. A schematic diagram of the welding setup is shown in Figure 2. The lap-joint structure of PC and PA66 was fixed in the welding fixture. A quartz glass plate with high transmittance at 1940 nm was placed on the PVC, while the PA66 layer rested on an aluminum plate. The welding trajectory followed a linear contour pattern, with the welding speed set at 1.5 mm/s and the defocus value fixed at 0 µm. The influence of different power levels on the welding quality of etched specimens was examined, and the optimal welding power under etching conditions was subsequently determined and applied to the other etched samples.

### 2.4. Characterization and Mechanical Property Testing

Following laser etching, the surface microstructure of the material significantly influences the welding performance. To clarify the bonding mechanism of dissimilar polymers, the surface morphology of laser-etched PVC was characterized using a laser scanning confocal microscope (LSCM). The analysis focused on etching quality, including the presence of residues or recast layers, the extent of thermal deformation, and the distinctness of boundary features. After welding, the internal microstructure of the weld and its cross-section were examined by optical microscope (OM) and scanning electron microscope (SEM) for localized observation. The fusion efficiency of the joint was subsequently evaluated, and the shear strength was determined. In parallel, the corresponding failure modes, namely interfacial failure, matrix failure, and mixed failure, were identified. Shear tests were performed using a universal testing machine, and the shear strength values were calculated according to Equation (1). Here, L is the length of the weld seam, which corresponds to the controllable laser scanning length, while B represents the actual effective bonding area width formed after laser transmission welding. After completing the welding experiment, the welded sample was placed under an optical microscope to measure the weld width B. In the calculation of welding strength, the B value for each experimental specimen is measured independently.

(1) σ=F/(L∗B)

In the equation, σ denotes the shear strength (MPa), F is the shear force (N), L is the weld seam length (20 mm, a fixed value), and B represents the weld seam width (mm).

## 3. Results and Discussion

### 3.1. Surface Morphology of Grooves Etched by Femtosecond Laser

**Figure 3:**
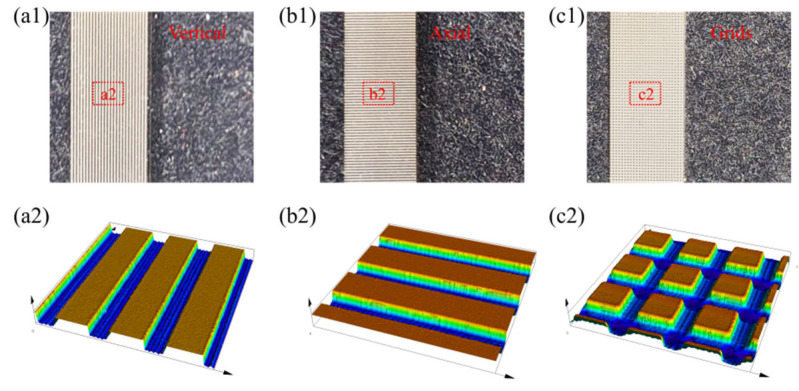
Different etching patterns: (**a1**,**a2**) vertical grooves; (**b1**,**b2**) axial grooves; (**c1**,**c2**) grid grooves (W = 70 μm, D = 70 μm, S = 130 μm).

Femtosecond laser etching was performed on PVC. Using a filled scanning strategy, vertical, axial, and grid grooves were fabricated on the PVC surface. The surface morphology was characterized by an LSCM to obtain three-dimensional topographic images (Figure 3). During ultrafast laser processing, the laser exhibits extremely high peak power, which induces instantaneous vaporization of the material within the irradiated region. Because the pulse width is shorter than the characteristic time of electron/phonon interaction, the laser energy deposited in the electron gas cannot be efficiently transferred to adjacent atoms or the lattice. Consequently, the deposited energy is carried away with the vaporized material, while the laser irradiation of this pulse simultaneously terminates. Ultrafast laser processing is characterized by a minimal heat-affected zone and sharply defined processing boundaries, resulting in regular morphology and distinct edges [33,34].

### 3.2. Influence of Groove Parameters on Welding Strength

#### 3.2.1. Influence of Groove Width

Current research on laser etching-modified welding of polymers has only focused on single vertical groove structures, without systematically establishing a relationship between microgroove geometric parameters and joint strength, nor exploring the influence of the matching between groove configuration and tensile loading direction on welding performance. To address this gap, this subsection employs the single-factor variable method to systematically investigate the effects of groove width, depth, spacing, and configuration on joint shear strength, determine the optimal microstructure parameters, and reveal the mechanism by which groove geometric features regulate interfacial bonding. All welding experiments in this subsection were conducted at a fixed welding power of 13 W (the optimality of this power will be verified in Section 3.3), strictly adhering to the single-variable principle.

**Figure 4:**
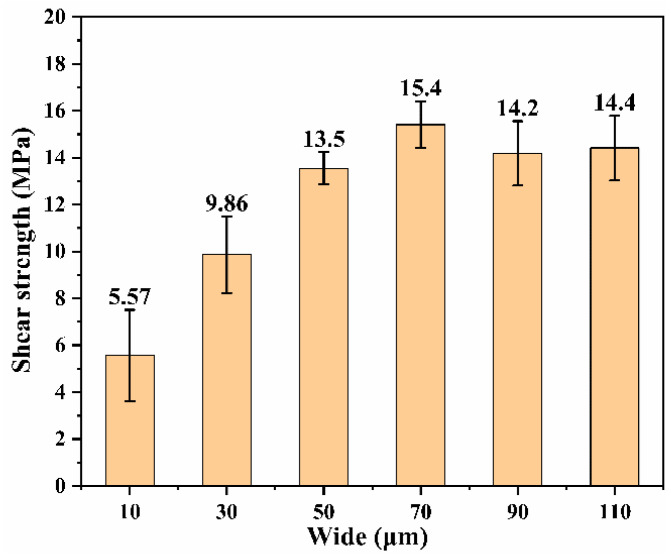
Variation of connection strength with increasing groove width (D = 70 μm, S = 130 μm).

**Figure 5:**
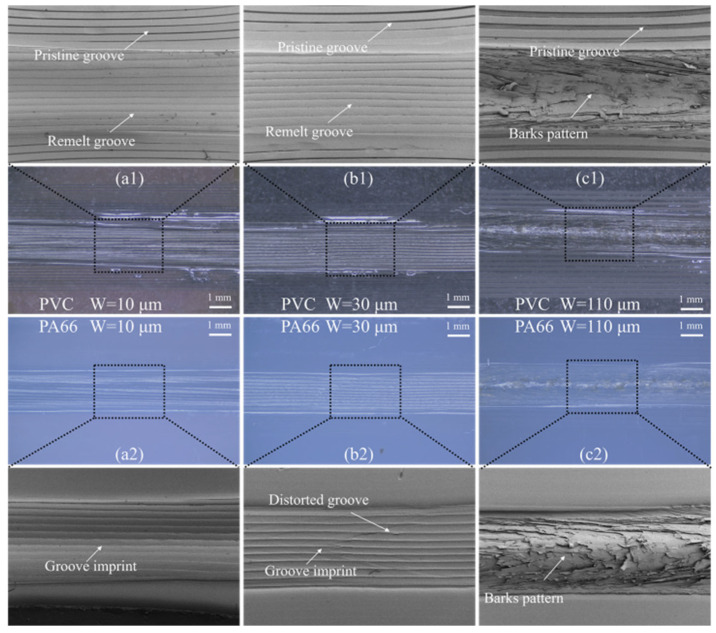
OM and SEM images of the fracture surfaces of joints with different groove widths. (D = 70 μm, S = 130 μm): (**a1**,**a2**) W = 10 μm; (**b1**,**b2**) W = 30 μm; (**c1**,**c2**) W = 110 μm.

Figure 4 shows the relationship between joint shear strength and slot width (W). Within the investigated range of 10–110 μm, the strength first increases and then slightly decreases with increasing slot width, reaching a maximum of 15.4 MPa at W = 70 μm, significantly higher than the 8.9 MPa achieved by femtosecond laser ablation treatment alone. Microscopic examination reveals that for failed joints with slot widths of W = 10 μm and W = 30 μm, the fracture surfaces of PVC and PA66 clearly preserve the slot-shaped structure (Figure 5), with only limited local remelting observed in a few slots (Figure 5(a1,a2). This indicates that when the slot width is too narrow, the PA66 melt possibly cannot adequately penetrate the slots, preventing the formation of an effective anchoring effect. Under fixed slot spacing, a smaller slot width also reduces the effective chemical bonding area in the weld seam, leading to a relatively low joint strength of 9.86 MPa. During the shear test, PVC and PA66 tend to separate at the interface, causing deformation of the slot structures on the PVC fracture surface, while the PA66 surface exhibits striated protrusions resembling indentations (Figure 5(a2)). In contrast, the fracture surfaces of the joint with W = 110 μm (Figure 5(c1,c2)) display distinct bark-like fractures. This suggests that a wider slot allows more PA66 melt to form effective anchoring with PVC, resulting in higher bonding strength (14.4 MPa). In summary, the joint strength exhibits a trend of rapid increase followed by a slight decline with increasing groove width. A maximum strength of 15.4 MPa is achieved when the width is increased to 70 μm; further increasing the width leads to a slight reduction in joint strength. Failed joint analysis reveals that narrow grooves result in low strength due to insufficient infiltration of PA66 melt and a relatively small effective bonding area, whereas excessively wide grooves reduce the tensile strength of the substrate due to excessive material removal. Thus, 70 μm is identified as the optimal groove width that balances joint strength and substrate performance.

#### 3.2.2. Influence of Groove Depth

**Figure 6:**
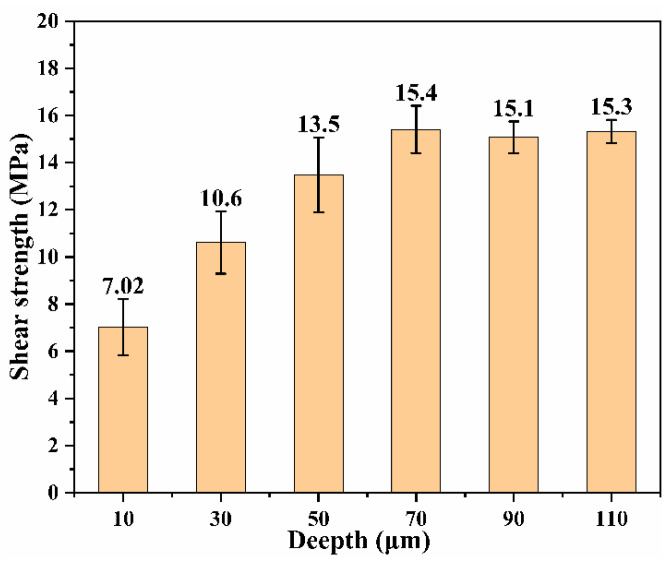
Variation of connection strength with increasing groove depth (W = 60 μm, S = 200 μm).

**Figure 7:**
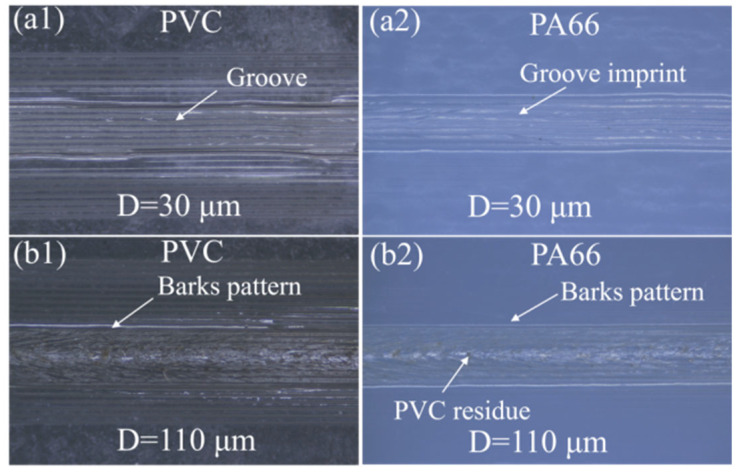
OM images of the fracture surfaces of joints with different groove depths (W = 60 μm, S = 130 μm): (**a1**,**a2**) D = 30 μm; (**b1**,**b2**) D = 110 μm.

**Figure 8:**
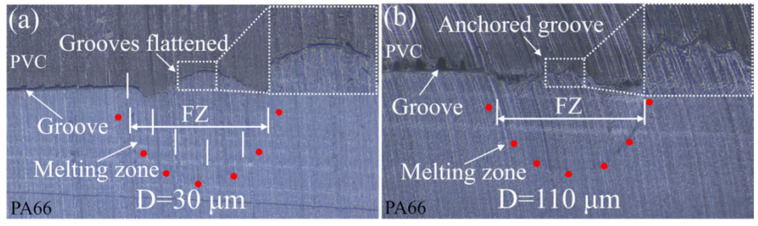
Micrographs of weld cross-sections with different groove depths (W = 60 μm, S = 130 μm): (**a**) D = 30 μm; (**b**) D = 110 μm.

When the groove depth is shallow (D ≤ 30 μm), the PA66 melt readily penetrates to the bottom of the PVC groove during welding. However, due to the absence of a pronounced interlocking mechanism between the dissimilar materials, the macroscopic anchoring structure formed within the weld seam provides only a limited contribution. As a result, the bonding strength of such hybrid joints remains low (≤10.6 MPa, Figure 6). The failure mode of these joints is mainly interfacial fracture (cf. Figure 7(a1,a2)). The optical microscopy (OM) image of the cross-section at D = 30 μm (cf. Figure 8a) shows that the PVC groove surface experienced heating and remelting, leading to a relatively smooth surface, which further weakened the macroscopic anchoring effect. In contrast, the cross-section of the welded joint at D = 110 μm (cf. Figure 8b) clearly reveals the formation of a pronounced macroscopic anchoring structure between the PVC and PA66. The PVC groove also underwent a marked morphological change, transforming from a rectangular to an inverted V-shape, with a significant increase in aspect ratio (defined as the maximum diameter-to-depth ratio). This transformation can be attributed to the penetration of PA66 melt into the groove opening and its continuous expansion under heating, which increased the groove diameter and remelting of the PVC-filled part of the groove bottom, thereby reducing the depth. Consequently, excessive groove depth does not markedly enhance the bonding strength. Specifically, the strength reaches 15.1 MPa at D = 90 μm and 15.3 MPa at D = 110 μm, with an extremely small increase. To conclude, the joint strength exhibits a distinct two-stage behavior with increasing groove depth. Within the range of 10 μm to 70 μm, the strength increases rapidly with depth, reaching a peak of 15.4 MPa. In contrast, when the depth is in the range of 70 μm to 110 μm, the strength only undergoes minor fluctuations. In addition, failed joint characterizations reveal that excessively shallow grooves fail to form effective macroscopic anchoring, while excessively deep grooves lead to morphological deformation and bottom filling by remelted material during welding, which not only fails to further improve the strength but also increases processing costs. Therefore, the optimal groove depth is 70 μm.

#### 3.2.3. Influence of Groove Space

**Figure 9:**
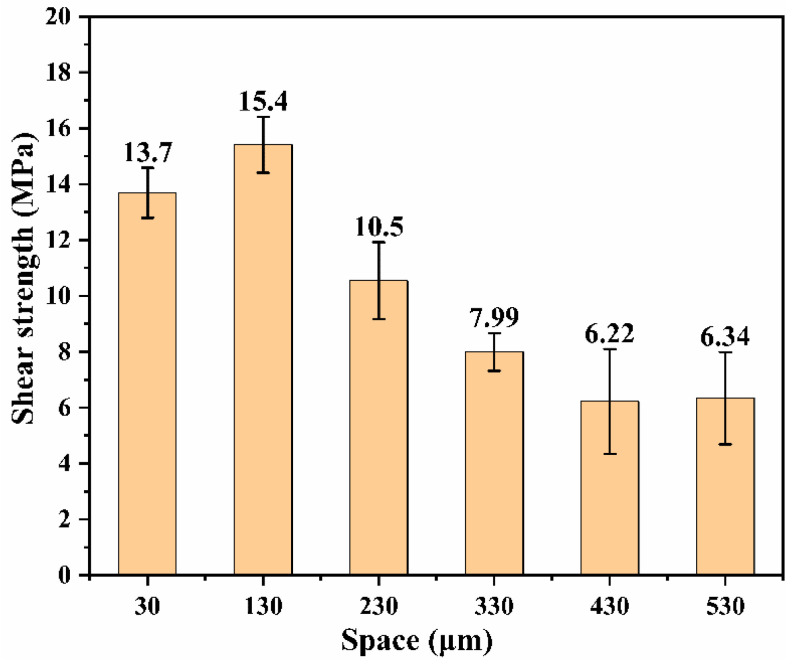
Variation of connection strength with increasing groove spacing (W = 60 μm, D = 60 μm).

**Figure 10:**
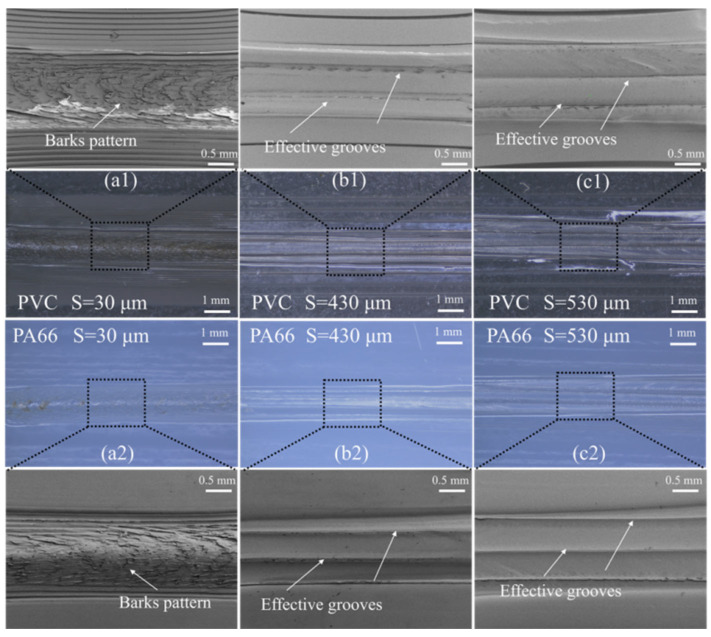
OM and SEM images of the fracture surfaces of joints with different groove spacing (W = 60 μm, D = 60 μm): (**a1**,**a2**) S = 30 μm; (**b1**,**b2**) D = 430 μm; (**c1**,**c2**) S = 530 μm.

As the groove spacing increases, the number of grooves contributing to the macroscopic anchoring mechanism diminishes. Consequently, when the spacing exceeds 230 μm, the shear strength of the joint gradually declines (Figure 9). At S = 430 μm and S = 530 μm, only 3–4 grooves form effective anchoring structures (Figure 10), resulting in relatively low connection strengths of 6.22 MPa and 6.34 MPa, respectively. Fracture surface analysis after shear testing shows protruding features on the PA66 surface that correspond to the PVC grooves (Figure 10), indicating an interfacial fracture mode. Although the specimen with S = 30 μm also exhibits bark-like tear features caused by shear (Figure 10(a1,a2)), its connection strength is lower than that of the specimen with S = 130 μm. This indicates that when the spacing is too small (S < 130 μm), the effective bearing area of PVC within the anchoring structure is insufficient, making it susceptible to tearing during shearing and thereby reducing joint strength. Moreover, smaller groove spacing increases the processing time of femtosecond etching. In summary, the joint strength exhibits a trend of initial increase followed by continuous decrease with increasing groove spacing, with a precipitous drop in strength occurring when the spacing exceeds 230 μm. Characterization of failed joints reveals that smaller spacing results in insufficient effective load-bearing area of PVC between grooves, rendering it susceptible to tearing, while larger spacing significantly reduces the number of effectively anchored grooves. Thus, a groove spacing of 130 micrometers was determined to be the optimal parameter.

#### 3.2.4. Influence of Groove Types

Current research has only conducted mechanical tests on single-groove structures perpendicular to the tensile loading direction, without investigating the matching relationship between groove orientation and loading direction, nor have they explored the performance of complex configurations such as grid grooves. As such, they fail to provide a design basis for multidirectional complex loading conditions. To address this gap, this subsection systematically compares the effects of three configurations—axial grooves, vertical grooves, and grid grooves—on joint shear strength, based on the optimal groove parameters (W = 70 μm, D = 70 μm, S = 130 μm) obtained from the aforementioned single-factor optimization experiments. Special emphasis is placed on analyzing the adaptability of different configurations to the tensile loading direction.

**Figure 11:**
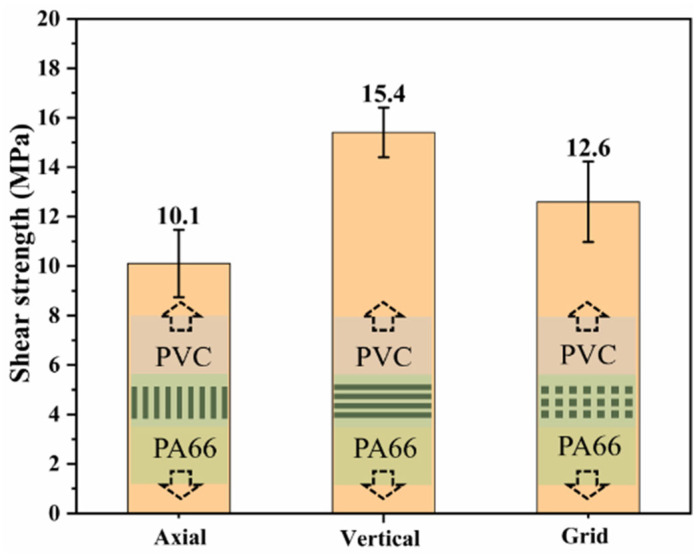
Variation of connection strength with diverse types of grooves.

**Figure 12:**
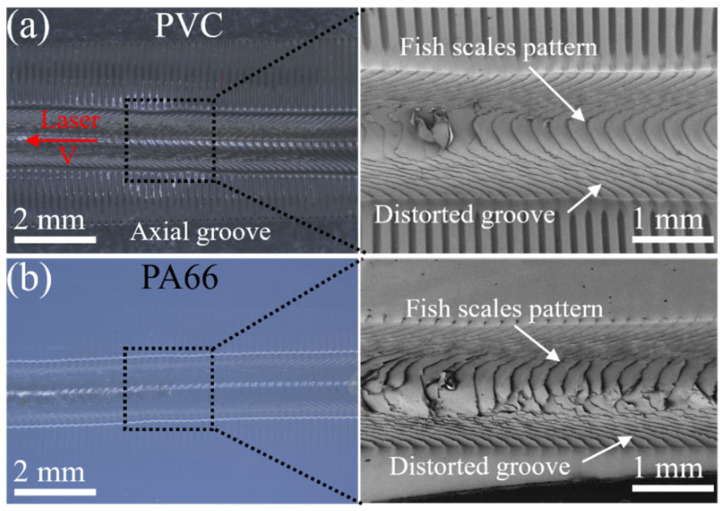
Image of the fracture surface of the failed joint with axial grooves: (**a**) PVC; (**b**) PA66.

To evaluate the influence of groove type on the bonding strength, various grooves were fabricated at the optimal laser power determined in the previous experiments, with groove depth and width corresponding to the condition of maximum welding strength. The measured bonding strengths are presented in Figure 11. Although the axial groove is aligned with the shear direction and is theoretically unfavorable for forming an effective anchoring structure, the joint still achieved a maximum average shear strength of 10.1 MPa. Failure analysis of the axial groove joint revealed that the PVC surface at the fracture exhibited a regular fish scale morphology (Figure 12). This feature is attributed to the flow of the molten pool along the laser scanning direction, which is perpendicular to the groove. This molten pool motion deforms the softened PVC groove, and subsequent solidification leads to the formation of the fish scale structure. However, axial grooves are parallel to the tensile loading direction and, thus, fail to form effective interlocking. 

**Figure 13:**
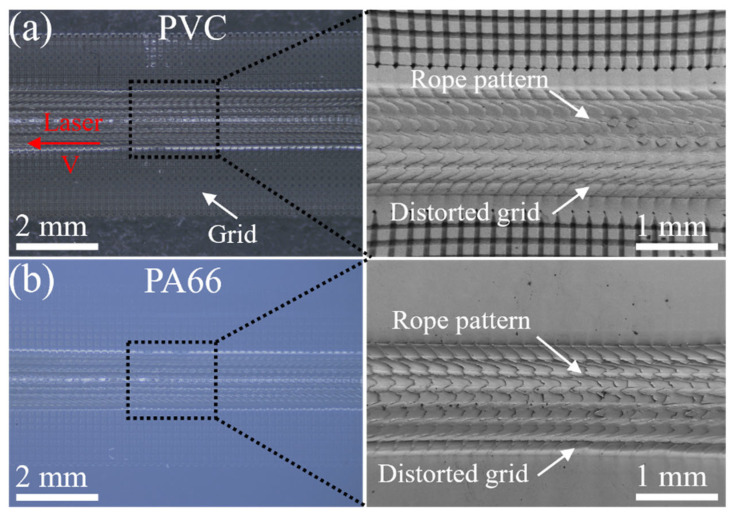
Image of the fracture surface of the failed joint with grid grooves: (**a**) PVC; (**b**) PA66.

Limited anchoring is achieved via groove deformation induced by molten pool flow, resulting in relatively low joint strength. In contrast, vertical grooves are perpendicular to the tensile loading direction, enabling stable mechanical interlocking that inhibits interfacial slip. Consequently, the maximum unidirectional strength reaches 15.4 MPa. Furthermore, grid groove joints enable anchoring in both axial and vertical directions simultaneously. While their unidirectional strength is slightly lower than that of vertical groove joints, with a maximum shear strength of 12.6 MPa, they address the critical limitation of unidirectional grooves being sensitive to tensile loading direction. Fracture analysis indicated that a rope-like tearing structure was formed at the weld center (Figure 13), suggesting the establishment of a high-strength anchoring structure between the dissimilar materials. Noticeable deformation was also observed at the weld edge of the grid structure, with part of the structure displaced along the laser scanning direction, indicating that molten pool motion also affects the grid geometry. Although the bonding strength of the grid groove joint is slightly lower than that of the vertical groove joint (approximately 2.8 MPa), it exhibits stronger adaptability to the shear direction. In summary, under the optimal groove geometric parameters, the maximum shear strengths of joints with axial grooves, vertical grooves, and grid grooves are 10.1 MPa, 15.4 MPa, and 12.6 MPa, respectively. Combining fracture analysis and force characteristic analysis, axial grooves parallel to the tensile direction only achieve limited anchoring via groove deformation induced by molten pool flow, resulting in the poorest strength performance. In contrast, vertical grooves perpendicular to the tensile direction enable stable mechanical anchoring to inhibit interfacial slip, ultimately yielding the optimal unidirectional shear strength. Meanwhile, the grid groove structure forms anchoring in two directions simultaneously; though its maximum unidirectional strength is slightly lower than that of vertical grooves, it addresses the critical limitation of unidirectional grooves’ sensitivity to loading direction, exhibiting superior adaptability to multidirectional complex loading conditions.

### 3.3. Influence of Laser Welding Power on Welding Strength

The aforementioned study determined the optimal geometric parameters of femtosecond laser microgrooves. As welding power is a core process parameter governing interfacial melting behavior, molten pool filling efficiency, and joint performance, this subsection employs specimens with the optimal vertical groove parameters (W = 70 μm, D = 70 μm, S = 130 μm) optimized in Section 3.2 to systematically investigate the influence of laser power on weld morphology, shear strength, and failure modes of the joints, thereby defining the optimal welding process window.

**Figure 14:**
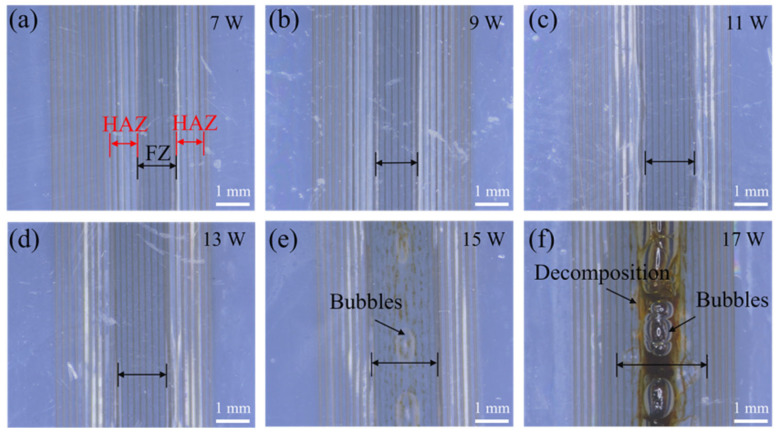
Micrographs of weld seams under different welding powers (W = 70 μm, D = 70 μm, S = 130 μm): (**a**) P = 7 W; (**b**) P = 9 W; (**c**) P = 11 W; (**d**) P = 13 W; (**e**) P = 15 W; (**f**) P = 17 W.

**Figure 15:**
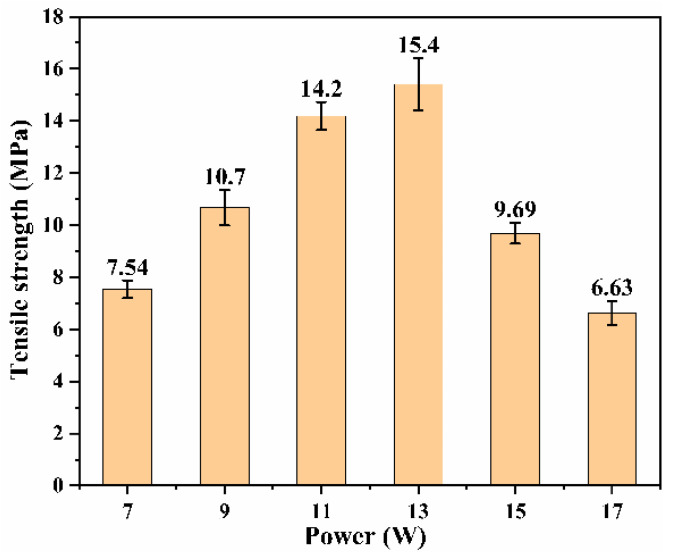
Variation of connection strength with increasing laser power (W = 70 μm, D = 70 μm, S = 130 μm).

To more accurately evaluate the influence of micro-grooves on welding strength, the effect of laser power on welding performance was first investigated to identify the optimal power parameter. Micrographs of welds produced under different laser powers are presented in Figure 14 (samples with etched vertical grooves). As the laser power increased from 7 W to 17 W, the weld seam width expanded from 1.35 mm to 2.62 mm. Correspondingly, the joint shear strength initially increased and then declined. At P = 13 W, the maximum shear strength of 15.4 MPa was obtained (Figure 15). When P > 15 W, the weld seam became significantly wider, and numerous bubbles appeared in its central region. These bubbles, generated by thermal decomposition, adversely affected both the airtightness and bonding strength of the weld seam. The weakening mechanism can be explained in two aspects. First, during shear loading, bubbles served as stress concentration sites. Second, they reduced the effective bonding area between dissimilar materials. At P = 17 W, polymer carbonization occurred, and a distinct yellow-black carbonized zone appeared in the center of the weld seam. Thus, when P > 15 W, the joint strength decreased markedly. Considering both the joint strength and weld appearance, 13 W was identified as the optimal laser power for etching treatment and was adopted as the standard parameter for subsequent welding experiments.

The maximum shear strength of the joints welded after laser etching reached 15.4 MPa, which was markedly higher than that of the joints welded after laser ablation (8.9 MPa). This result indicates that the improvement in compatibility caused by laser modification alone cannot explain the substantial increase in bonding strength observed after laser etching, implying the involvement of additional non-chemical bonding mechanisms. To clarify the bonding mechanism, shear tests were conducted on the joints, and the microstructure of the weld seams was examined using optical microscopy to identify the failure modes of tensile fracture.

**Figure 16:**
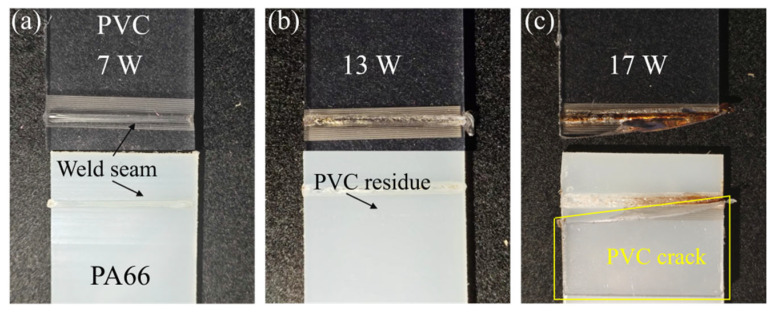
The fracture failure modes of the joints under different laser power: (**a**) interface; (**b**) mixture; (**c**) PVC matrix.

**Figure 17:**
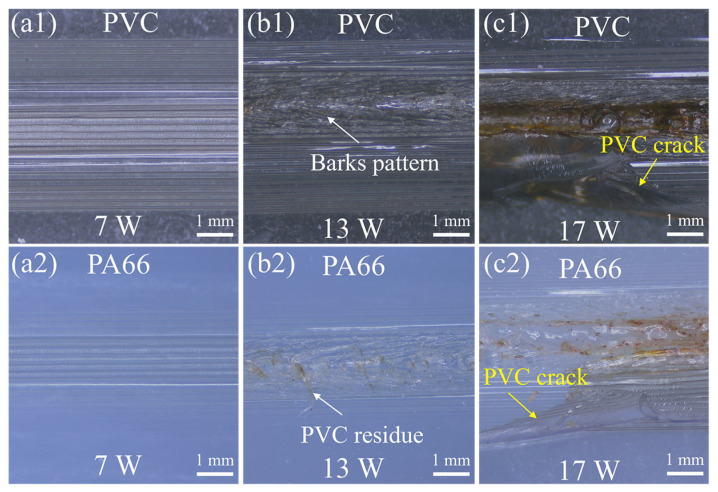
OM images of joint fracture surfaces at different powers (W = 70 μm, D = 70 μm, S = 130 μm): (**a1**,**a2**) P = 7 W; (**b1**,**b2**) P = 13 W; (**c1**,**c2**) P = 17 W.

The shear failure specimens obtained under different welding powers and their corresponding microstructures are presented in Figure 16 and Figure 17. Because PA66 exhibits a relatively high absorption rate, it is heated and melted first during welding. The resulting thermal expansion allows it to adequately fill the PVC grooves, thereby forming an anchoring structure. At a low laser power of 7 W, PVC fails to reach a molten state due to insufficient heat input, leading to limited molecular diffusion and entanglement between PVC and PA66. Consequently, the bonding strength of the joint is low, only 7.54 MPa. Microscopic observation of the fracture surface after shear testing shows that the groove structure on the PVC surface remains intact (Figure 17(a1)), indicating interfacial fracture at the weakly bonded interface. When the welding power exceeds 13 W, sufficient heat accumulates on the PVC surface to enable melting, allowing the molecules of both materials to diffuse and entangle effectively. This results in the formation of a dense macroscopic anchoring structure in the fusion zone, significantly enhancing the bonding strength (15.6 MPa). In the high-strength joint at P = 13 W, shearing caused tearing of the material along the weld seam, with the PVC surface exhibiting a bark-like fracture morphology (Figure 17(b1)), while distinct tear marks were also observed on local regions of the PA66 surface (Figure 17(b2)). This indicates a mixed-fracture failure mode during shearing. When the power exceeds 15 W, continuous heat accumulation at the interface causes local temperatures to approach and exceed the initial thermal decomposition temperature of PVC, leading to molecular chain degradation and bubble-like defects. At even higher power levels, PVC undergoes noticeable discoloration and carbonization, significantly reducing the matrix’s inherent strength. These thermal decomposition products not only act as stress concentration sites that trigger interfacial cracking but also reduce the effective bonding area and disrupt the mechanical interlocking structure, ultimately resulting in a rapid decline in joint strength. Under these conditions, the tensile strength of the PVC matrix decreases to a level below the bonding strength of the joint, ultimately reducing the overall joint strength and resulting in matrix fracture failure (Figure 16c).

Overall, the femtosecond laser enables the fabrication of three types of microgrooves (vertical, axial, and grid) with regular morphology and well-defined edges on the PVC surface. Subsequently, in laser transmission welding experiments, single-factor variable experiments were conducted to determine the optimal groove parameters: width of 70 μm, depth of 70 μm, and spacing of 130 μm. Among these, grooves perpendicular to the tensile loading direction yielded the optimal unidirectional shear strength. In contrast, grid microgrooves, despite slightly lower unidirectional strength, exhibited superior adaptability to multidirectional loading conditions. Finally, 13 W was identified as the optimal welding power, corresponding to a maximum joint shear strength of 15.4 MPa, achieving high-strength welding between PVC and PA66.

### 3.4. Analysis of Strengthening Mechanism

The above experimental results demonstrate that femtosecond laser microstructuring can significantly enhance the joint strength of the immiscible PVC/PA66 system, and there are notable differences in reinforcement effects and load adaptability among different groove configurations. To reveal its internal strengthening mechanisms, this section elucidates the mechanical interlocking effect and stress regulation patterns of micro-groove structures through fracture surface morphology analysis and finite element numerical simulation, verifying the adaptability advantages of grid grooves under multidirectional loading.

To further clarify the effect of groove geometry on welding strength, ANSYS software was employed to simulate the tensile strength test of welded specimens subjected to different treatments under identical tensile loads. In the simulation, an ideal elastic–plastic constitutive model was adopted, and the input room temperature mechanical property parameters are as follows: the Young’s modulus of PVC is 2.62 GPa with a Poisson’s ratio of 0.37, while the Young’s modulus of PA66 is 2.9 GPa with a Poisson’s ratio of 0.34. The 3D joint model was discretized using a hexahedral structured mesh. The global mesh element size was set to 40 µm, and the feature suppression size was kept at the system default value of 10 µm to ensure the accurate capture of fine geometric features such as micro-grooves. Meanwhile, local mesh refinement was performed on critical stress concentration regions, including the weld interface and micro-grooves. After refinement, the minimum element size at the sidewalls of micro-grooves and the interface was 10 µm, while the bulk matrix regions away from the interface retained the global mesh size of 40 µm to balance computational accuracy and efficiency. All external surfaces of the model are subjected to directional displacement constraints, restricting translational degrees of freedom in the Y and Z directions as well as all rotational degrees of freedom, while only allowing translational freedom in the X direction. Subsequently, an axial working load is applied along the X direction, enabling the structure to undergo only unidirectional motion and deformation in the X direction. A bonded contact interface is used to simulate the strong bonding state after welding.

**Figure 18:**
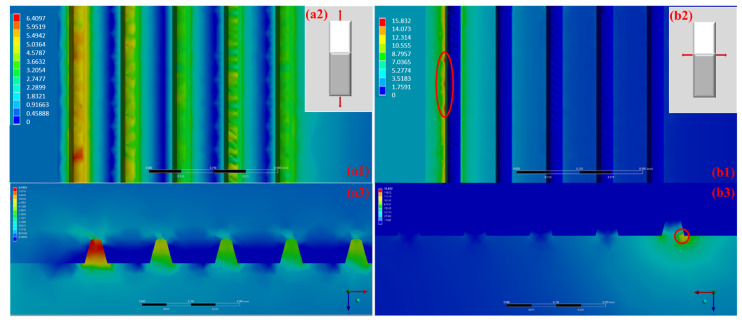
Stress simulation analysis of tensile forces in different directions of axial grooves: (**a1**–**a3**) tensile force is perpendicular to the groove direction; (**b1**–**b3**) tensile force is parallel to the groove direction.

Figure 18 presents the simulated internal stress distribution within the axial groove during the tensile test, where (a1)/(b1) represent the top view and (a3)/(b3) represent the front view of the tensile surface. The loading directions are shown in Figure 18(a2,b2), with tensile forces of equal magnitude applied in different orientations. When the tensile force is perpendicular to the groove direction, the maximum internal stress at the first groove reaches 6.41 MPa, while the stresses in the remaining grooves are uniformly distributed. In contrast, when the tensile force is parallel to the groove direction, the internal stress is concentrated at a single point on the edge of the first groove, reaching a peak value of 15.83 MPa. Under identical tensile loading, parallel alignment between the groove and tensile force results in localized stress concentration, leading to earlier yielding and fracture of the specimen at a relatively lower tensile force. Thus, specimens with grooves oriented perpendicular to the tensile direction exhibit superior strength.

**Figure 19:**
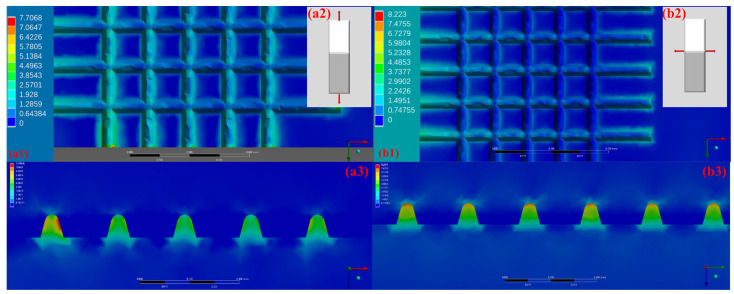
Stress simulation analysis of tensile forces in different directions of grid grooves: (**a1**–**a3**) tensile force is perpendicular to the welding direction; (**b1**–**b3**) tensile force is parallel to the welding direction.

Figure 19 presents the simulated internal stress distribution for specimens with axial grooves under tensile loading, where (a1)/(b1) are the top views and (a3)/(b3) are the front views of the tensile surface. The loading directions are given in Figure 19(a2,b2), with identical magnitudes in each case. The results indicate that under tensile loading in different directions, the internal stresses are consistently lower than those presented in Figure 18(b1) and are more uniformly distributed. This demonstrates that grid grooved specimens reduce internal stress regardless of loading orientation, thereby improving welding strength. Considering the variability of tensile force directions in engineering practice, grid grooves are more suitable for practical applications than axial grooves.

**Figure 20:**
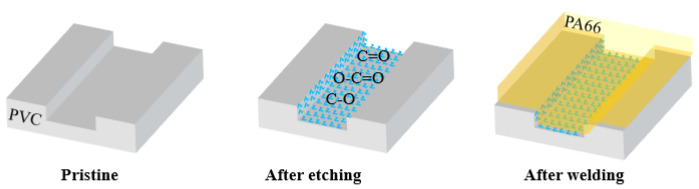
Schematic diagram of laser etching and connection mechanism.

As depicted in Figure 20, the mechanism by which femtosecond laser surface etching facilitates the formation of high-strength joints can be summarized in two aspects. First, femtosecond laser etching introduces many oxygen-containing functional groups onto the PVC surface, which enhances interfacial compatibility, mitigates the effect of the weak boundary layer (WBL), and improves bonding performance [9,19]. Second, and more importantly, the grooves formed by laser etching provide a favorable macroscopic anchoring effect for welding dissimilar materials. The combined contribution of these two factors—enhanced bonding performance and macroscopic anchoring—significantly increases the joint strength of incompatible PVC and PA66 materials.

## 4. Conclusions

In response to the challenges posed by the inadequate welding performance of dissimilar polymers with poor mutual solubility, as well as the limitations of traditional surface modification techniques, this research proposes the use of femtosecond laser surface etching technology to enhance welding efficacy. To achieve this goal, a comprehensive series of experiments was designed, including femtosecond laser etching of polymers, mid-infrared LTW of dissimilar polymers, mechanical property assessments, and characterization. Initially, PVC was subjected to femtosecond laser surface treatment using various etching parameters. Following this, the surface-treated PVC/PA66 specimens were welded using mid-infrared lasers at different power levels. The mechanical properties of the composite joints were then evaluated to determine the optimal welding power and etching conditions. Based on the experimental results, both etching and welding outcomes were assessed. Furthermore, an in-depth analysis was conducted to explore the mechanisms underlying the improved welding performance of dissimilar polymers after etching. This methodology provides an efficient and reliable joining solution for the PVC/PA66 system, which is widely used in industry but notoriously difficult to weld due to poor compatibility. Characterized by non-contact processing and the absence of chemical contamination, this technique effectively mitigates the thermal decomposition issue of PVC. Additionally, it offers high parameter controllability and facile integration into existing automated production lines. Furthermore, the revealed matching law between micro-groove configurations and load directions in this study provides a theoretical basis for the structural design of polymer joints under complex working conditions, and holds profound significance for advancing the engineering application of femtosecond laser welding technology in the field of incompatible polymer joining.

The key experimental findings and conclusions are presented as follows.

(1) To address the critical challenge of weak interfacial adhesion and low joint strength in laser welding of incompatible PVC/PA66 dissimilar polymers, this study proposes a welding strengthening strategy via femtosecond laser-induced surface microstructuring. This approach enables the fabrication of high-precision, controllable microgroove structures on the PVC surface, thereby significantly enhancing the joint welding strength.

(2) When a vertical groove structure (width = 70 μm, depth = 70 μm, spacing = 130 μm) is employed with a welding power of 13 W, the maximum shear strength of the joint reaches 15.4 MPa—significantly higher than the 8.9 MPa achieved by femtosecond laser ablation treatment alone.

(3) Vertical microgrooves deliver the optimal unidirectional shear strength, whereas grid microgrooves effectively mitigate stress concentration under multidirectional loading conditions. They exhibit superior adaptability to complex stress states, addressing the critical limitation of traditional unidirectional grooves being sensitive to loading direction.

However, this study only investigated the joint performance under room temperature static shear loading, and long-term reliability tests under complex environments, such as fatigue, high temperature, and hygrothermal aging, remain unconducted. Future research could carry out systematic studies to address the aforementioned limitations, while extending this technology to a broader range of incompatible polymer systems, thereby further refining the theoretical system and engineering application foundation of femtosecond laser microstructured welding.

## Figures and Tables

**Table 1:** Surface treatment processes and welding performance of dissimilar polymers and their composite materials.

**Materials**	**Treatment Methods**	**Bonding Methods**	**Bonding Strength**		**Years**
			**Before Treatment**	**After Treatment**	
PE/PA66	Grafted maleic anhydride on PE	Laser transmission welding	0	~10.7 MPa	2016 [14]
PA66/PC	Cold spraying Al on PA66GF20	Laser transmission welding	0	~4.0 MPa	2016 [15]
PA66/PVC	Magnetron sputtering Al on PA66	Laser transmission welding	0	~4.2 MPa	2017 [16]
TPE/PMMA	O_2_ plasma treatment on TPE	Thermal bonding	~4.5 MPa	~16.5 MPa	2017 [17]
PE/POM	Plasma surface treatment on PE	Laser transmission welding	0	~6.1 MPa	2018 [10]
PC/PS	Iron powder particles absorbes	Laser transmission welding	0	~4.12 MPa	2024 [18]
PC/PS	Femtosecond laser surface treatment	Laser transmission welding	0	~13.65 MPa	2024 [19]

**Table 2:** Laser surface etching parameters.

**Parameter**	**Data**					
groove width/μm W/(D = 60 μm, S = 200 μm)	10	30	50	70	90	110
groove depth/μm D/(W = 60 μm, S = 200 μm)	10	30	50	70	90	110
groove space/μm S/(W = 60 μm, D = 80 μm)	30	130	230	330	430	530
types of grooves	Axial	Vertical	Grids			

## Data Availability

The original contributions presented in this study are included in the article. Further inquiries can be directed to the corresponding author.
